# Systematic Curiosity as an Integrative Tool for Human Flourishing: A Conceptual Review and Framework

**DOI:** 10.1007/s12124-024-09856-6

**Published:** 2024-07-09

**Authors:** Anne-Laure Le Cunff

**Affiliations:** 1 Institute of Psychiatry, Psychology & Neuroscience, King’s College London, London, SE5 8AB UK; 2 Ness Labs, 40 Downham Road, London, N1 5AL UK

**Keywords:** Curiosity, Flourishing, Psychology, Lifespan Development

## Abstract

This paper reviews seventy years of theoretical research and proposes systematic curiosity as an integrative tool for human flourishing with a focus on four key aspects: firstly, acknowledge curiosity’s multidimensional nature instead of harmonizing its complex taxonomy; secondly, emphasizing intentional curiosity as opposed to impulsive curiosity; thirdly, prioritizing domain-general curiosity for broader applicability across educational, organizational, and therapeutic settings; and lastly, focusing on curiosity as a developable skill rather than an innate trait. By segmenting systematic curiosity into cognitive, emotional, and behavioral components, and relating these to interactions with the self, others, and the world, the framework aims to apply across the spectrum of human experience. Furthermore, the framework encourages an exploration of various evidence-based activities for flourishing so individuals can discover the most suitable strategies for their specific context. Implications for both theory and practice are examined, limitations are discussed, and avenues for future research are suggested.

**Supplementary Information:**

The online version contains supplementary material available at 10.1007/s12124-024-09856-6.

## Introduction

Flourishing has been defined as “[living] within an optimal range of human functioning, one that connotes goodness, generativity, growth, and resilience” (Fredrickson & Losada, 2005). Historically rooted in philosophical traditions, flourishing has evolved from its ancient conceptualization in Aristotelian ethics, where it was synonymous with a life well-lived, to a more nuanced construct in contemporary research (Ryff, 2023; Waterman, 1990). This evolution reflects a shift from purely moral qualities to include psychological and emotional well-being (Narvaez, 2008, 2015). Flourishing is now thought to extend beyond happiness to encompass intellectual fulfillment, emotional resilience, and social contribution (Keyes, 2002; Ryan et al., 2008; Ryff, 1989). This broader view aligns with research suggesting that flourishing is not merely the absence of adversity but the presence of indicators of holistic development across multiple domains of human life (Schotanus-Dijkstra et al., 2016). As such, flourishing is seen as a holistic measure of success, transcending traditional metrics of achievement to include mental health, emotional regulation, and the ability to form constructive social relationships, and has recently been characterized as the fundamental aim of education (Kristjánsson, 2017, 2023).

The existence of a list of objective goods central to flourishing remains the subject of debate. Some scholars argue for universal criteria that define a flourishing life, while others advocate for a more subjective, individualized approach (Rice, 2013; Schinkel et al., 2023; Wolbert et al., 2018). Despite this, there is consensus on certain individual factors being integral to flourishing (Keyes, 1998; Levin, 2021; Ryff & Keyes, 1995). These include not only autonomy and achievement but also engagement in meaningful activities, the pursuit of knowledge, and the expression of one’s creativity and imagination (Conner et al., 2018; Kiverstein & Miller, 2023; Rice, 2013; Schinkel et al., 2023). These factors highlight the importance of personal choice and self-directed behavior in achieving a state of flourishing and reflects a growing understanding that human development is multidimensional.

Curiosity is also increasingly recognized as a key contributor in human flourishing (Whitecross et al., 2023). Curiosity has been described as “the impulse towards better cognition” by philosopher and psychologist William James (1899). Cicero described curiosity as a natural inclination towards acquiring knowledge for its own sake, without any expectation of gain, but he also saw it as an uncontrollable impulse, referring to Ulysses and the Sirens as a parable for curiosity (Cicero, 1998; Lowenstein, 1994). In the 19th century, David Hume (1888) already divided curiosity into two different categories: a positive type, which he referred to as a love of knowledge, and a negative type, which he considered a passion. He saw good curiosity as the kind that drives scientific research, while bad curiosity was viewed as an excessive eagerness to know the lives and affairs of others (Hume, 1888).

Moving away from these moral considerations, modern techniques have allowed researchers to probe further into the nature of curiosity. From a neurophysiological standpoint, curiosity is strongly linked to the brain’s reward system (Kidd & Hayden, 2015). Studies have shown that certain brain regions, such as the caudate nucleus and the inferior frontal gyrus, become active when individuals feel curious, implying that these areas play a role in the anticipation of the rewarding nature of acquiring new information, even though this anticipation does not always align with traditionally expected reward pathways, as indicated by the lack of activity in the nucleus accumbens (Kang et al., 2009). Further investigation suggested that states of high curiosity enhance memory retention, even for information that is not the focus of curiosity, highlighting the broad impact of curiosity on cognitive processes (Gruber et al., 2014). Additionally, the role of dopamine neurons in signaling both primary and informational rewards underlines the integral part of the dopaminergic reward system in mediating the experience of curiosity (Bromberg-Martin et al., 2010; Bromberg-Martin & Hikosaka, 2011). These findings collectively indicate that curiosity, while intrinsically motivated, shares common mechanisms with extrinsically motivated rewards, suggesting a complex interplay in how the human brain processes the desire for knowledge and new experiences.

From a psychological standpoint, curiosity is thought to motivate learning by spurring exploratory behaviors aimed at resolving gaps in knowledge (Gureckis & Markant, 2012; Oudeyer & Smith, 2016). As such, it fosters sense-making from childhood onwards (Kidd & Hayden, 2015). Whether through simple observation or active information-seeking, satisfying curiosity enables deeper encoding and retention of newly learnt information (Kang et al., 2009). Infants display exploratory behaviors such as gazing longer at unexpected events or novel stimuli that suggest early curiosity-driven learning (Mather, 2013; Stahl & Feigenson, 2015). As children develop cognitively, curiosity prompts question-asking and independent discovery of their physical, mental, and social world, facilitating cognitive and social development (Chouinard et al., 2007; Frazier et al., 2009; Jirout & Klahr, 2012; Liquin & Lombrozo, 2020). In adulthood, curiosity is considered an important trait for continuous learning and adaptability (Cronin-Golomb & Bauer, 2023; Sakaki et al., 2018; Fry et al., 2023).

Daily levels of curiosity have a significant impact on happiness and personal growth (Kashdan & Steger, 2007; Lydon-Staley et al., 2020; Reio & Sanders-Reio, 2020). Extensive research indicates that individuals with high curiosity exhibit more growth-oriented behaviors, derive a greater sense of meaning from life, and generally report higher levels of life satisfaction (Jovanovic & Brdaric, 2012; Kashdan & Steger, 2007; Peterson et al., 2007; Whitecross et al., 2023). They exhibit fewer symptoms of anxiety, depression, and burnout (Denneson et al., 2017; Garrosa et al., 2017; Kashdan et al., 2011). In professional settings, curiosity correlates positively with innovation (Celik et al., 2016) and is associated with increased job satisfaction and engagement (Kashdan et al., 2020). Furthermore, in educational contexts, a strong sense of curiosity is related to superior academic performance (Von Stumm et al., 2011). Curious individuals tend to pay more attention to their conversation partners and are more adept at discerning others’ personalities (Hartung & Renner, 2011). Curiosity is also linked to increased creativity (Schutte & Malouf, 2020), a quicker learning pace (van Schijndel et al., 2018), and enhanced memory retention (Padulo et al., 2022). These findings have led researchers to theorize that curiosity is a vital source of psychological well-being (Denneson et al., 2017; Garrosa et al., 2017; Whitecross et al., 2023).

The significance of curiosity in positively affecting various factors relevant to flourishing is evident, yet existing research has predominantly focused on delineating curiosity’s multifaceted nature or fostering curiosity as a general construct purported to support education, overlooking the potential applications of fostering curiosity to directly support flourishing (Arnone, 2003; Kashdan & Fincham, 2004; Pluck & Johnson, 2011). As a result, there is a lack of pragmatic tools that actively leverage this trait for holistic human development. This paper seeks to bridge this gap. Rather than attempting to reconcile the numerous existing typologies of curiosity, it seeks to identify and operationalize a type of curiosity that can be nurtured, practiced, and controlled. The aim is to develop an actionable framework that has tangible implications for basic research and real-world applications in fostering flourishing through the practice of curiosity.

## Methods

Conceptual reviews are considered appropriate when the aim is to “untangle” terms used in research and practice and to revisit existing research to develop and refine theory (Hulland, 2020; Schreiber & Cramer, 2022). A comprehensive literature search was conducted using the Web of Science, one of the largest multidisciplinary databases of research publications (Birkle et al., 2020), focusing on the term “curiosity” in conjunction with associated terms such as “framework,” “model,” “theory,” and “construct,” as well as variations of these terms (Appendix 1). This initial search yielded a total of 1,375 records. To examine theoretical discussions of curiosity, a filter was applied to select only review articles, which narrowed down the results to 92 records. The remaining records were screened for relevance, resulting in a set of 22 theoretical papers (Appendix 2). A synthesis matrix was created to summarize the results (Ingram et al., 2006). The synthesis matrix formed the basis for a framework conceptualizing aspects of curiosity that can be operationalized to support human flourishing (Ravitch & Riggan, 2016). The conceptual framework was built by mapping the selected data sources, extensively reading and categorizing the selected data, identifying and naming concepts, deconstructing and categorizing the concepts, integrating the concepts, and finally synthesizing the concepts into an integrated framework (Jabareen, 2009).

## Results

Despite the results spanning seventy years, more than 70% (*n* = 15) of the reviews exploring theoretical frameworks and operational definitions of curiosity were conducted in the past decade (2013–2023), indicating a growing interest in this area of research.

Early reviews focus on what can be described as impulsive curiosity, aligned with Hull’s drive theory (1943) which posits that behaviors are primarily motivated by the need to address biological needs and increase an organism’s potential for survival (Simpson & Balsam, 2016). Within this framework, curiosity is conceptualized as a motivational state that is aroused by specific types of stimuli (Berlyne, 1954). Curiosity motivates information-seeking, similar to how hunger motivates eating (Lowenstein, 1994). It is considered an adaptive function which contributes to survival, learning, and development (Voss & Keller, 1983). Those views emphasize the role of stimulation and arousal in exploratory behavior, proposing that these factors significantly influence the nature and extent of curiosity-driven actions (Lester, 1968). Already, researchers start to distinguish between curiosity as a temporary state and as a more enduring personality trait (Boyle, 1983).

Departing from the early theories centered on drive and optimal arousal, modern research is now exploring the multifaceted nature of curiosity. Some researchers chose to focus on one dimension of curiosity. In particular, intellectual curiosity has been defined as a motivational state of cognitive stimulation that leads to exploratory behavior to acquire new knowledge or clarity in understanding, influenced by both internal desires and external contexts, with newly gained knowledge motivating further curiosity in an iterative process of inquiry (Russell, 2013). Other researchers have attempted to create more comprehensive models that encompass various aspects of curiosity. For instance, curiosity has been conceptualized as an intrinsic desire to explore the unknown, with recent research suggesting it is based on dimensions of *novelty* and *familiarity*, focusing on how frequently and in what contexts stimuli are encountered, rather than being goal-directed (Modirshanechi et al., 2023). Another framework combines the interest/deprivation model with the neuroscience of *wanting* and *liking* to provide a more comprehensive understanding of curiosity as an emotional-motivational state (Litman, 2005; Litman & Jimerson, 2004). Researchers have also proposed a neurological model of curiosity called the Prediction, Appraisal, Curiosity, and Exploration (PACE) framework which posits that curiosity arises from significant prediction errors, driving exploratory behaviors that bolster memory encoding, with dopamine neuromodulation providing further consolidation for memories formed during curious states (Gruber & Ranganath, 2019).

The results also indicate that the operationalization of curiosity is still a subject of debate. Researchers have proposed a new information-gap based measurement procedure focused on assessing young children’s scientific curiosity through exploratory behavior, defining curiosity as “the threshold of desired uncertainty in the environment that leads to exploratory behavior” (Jirout & Klahr, 2012). Other researchers operationalize curiosity through a “learning progress” (LP) signal that rewards activities leading to improvement in predictions and problem-solving over time, avoiding the need to define absolute uncertainty levels (Gottlieb et al., 2013). The LP hypothesis proposes that experiencing learning progress in an activity triggers intrinsic reward and curiosity, forming a positive feedback loop where curiosity drives activities that maximize further learning progress, which in turn enhances curiosity for those activities; this feedback loop causes learners to automatically focus on learnable activities tuned to their competence levels, progressing from simple to complex skills in a self-organized developmental trajectory (Oudeyer et al., 2016).

Yet more recent perspectives characterize curiosity as the practice of actively building one’s knowledge networks (Zurn & Bassett, 2018). This involves seeking out new information, forging connections between novel inputs and existing schemata, and expanding the scope and complexity of one’s mental representations. The very act of gathering and integrating new facts and experiences to resolve gaps strengthens and reorganizes knowledge networks. As such, curiosity manifests through exploratory behaviors aimed at sense-making—including manipulating novel objects, asking questions, or pursuing abstract ideas that provide learning opportunities (Spielberger & Starr, 2012).

One review (Grossnickle, 2014), in an attempt to disentangle the concepts of curiosity and interest, created an extensive map of the various definitions and dimensions of curiosity proposed in academic research: the dimensions can be based on the object of curiosity (physical, sensory, social, or epistemic), the breadth and the depth of curiosity, the degree of stability (state versus trait curiosity), the reason for curiosity (specific versus diversive, interest versus deprivation). However, disentangling curiosity and interest may not be necessary: it has been proposed that focusing on the subjective reward of gaining knowledge can advance understanding of sustainable exploration without needing definitive concepts of curiosity and interest (Murayama et al., 2019). Similarly, researchers have argued that progress in understanding curiosity’s biological and neural underpinnings has been hindered by an excessive focus on defining and categorizing it (Kidd & Hayden, 2015). They advocate for a more inclusive approach to studying curiosity, integrating it with other cognitive processes to gain deeper insights.

Instructional models suggest that curiosity flourishes when students take responsibility for learning, consider multiple perspectives, and reflect both on the subject matter and the learning process, which certain teaching strategies such as inquiry-based learning and group discussion can encourage (Dyche & Epstein, 2011). Researchers suggest that curiosity involves two key metacognitive monitoring steps: evaluating one’s own informational needs and predicting the likelihood of significant information gains from exploring the environment; this perspective indicates that curiosity can be procedurally deployed even by young children (Goupil & Proust, 2023). Curiosity training has been defined as “a process whereby individuals engage in the practice of certain mental states of curiosity” (Zurn & Bassett, 2018). It has also been argued that defining curiosity specifically within an academic domain, tracking students’ knowledge and behaviors as they advance, better distinguishes curiosity from interest and can help inform instructional interventions (Peterson & Cohen, 2019).

Cultivating curiosity in work environments shows promise as a way to support performance. A review found that organizational manifestations of curiosity such as establishing curiosity as a norm can increase creativity, adaptability, and learning, leading to higher job performance, job satisfaction, and well-being (Lievens et al., 2022). One model examined the role of curiosity in volatile, uncertain, complex, and ambiguous (VUCA) work environments, arguing that curiosity is associated with openness, exploration, a desire for knowledge, and a high tolerance to disruption—all attributes that can help employees adapt to change (Horstmeyer, 2020). However, researchers analyzed 13 curiosity measures and found limited application in organizational studies (only nine studies using five measures), highlighting a gap between the growing curiosity research literature and its practical use in organizations (Wagstaff et al., 2021).

In summary, despite the rich history of curiosity research spanning over seventy years, there remains a lack of consensus on its precise definition and operationalization. Nevertheless, contemporary research has increasingly acknowledged the multidimensional character of curiosity and its potential to support flourishing across a number of attributes (Table 1).

**Table 1:** Synthesis matrix of theoretical frameworks of curiosity

Attributes	Characteristics
*Dimensions*	• Curiosity should be understood from both a psychological and a neurological perspective (Gruber & Ranganath, 2019; Kidd & Hayden, 2015; Litman, 2005). • Curiosity can be physical, sensory, social, epistemic (Grossnickle, 2014; Russell, 2013). • Curiosity can be a temporary state and or an enduring personality trait (Boyle, 1983; Grossnickle, 2014). • Curiosity and interest may be overlapping phenomena (Grossnickle, 2014; Murayama et al., 2019).
*Drive*	• Shift from curiosity seen as impulsive in alignment with drive theory to a multifaceted view of motives driving curiosity (Berlyne, 1954; Hull, 1943; Lester, 1968; Loewenstein, 1994; Modirshanechi et al., 2023; Voss, 1983). • Curiosity can be driven by an information-gap based and maintained by a signal or learning progress (Jirout & Klahr, 2012; Gottlieb et al., 2013; Oudeyer et al., 2016). • Curiosity’s drive can be defined as actively building one’s knowledge networks “where one could purposefully leap from their existing knowledge network into an external pool of the knowledge network” (Zurn & Bassett, 2018).
*Domains*	• Curiosity plays an important role in learning, knowledge acquisition, and educational settings (Dyche & Epstein, 2011; Goupil & Proust, 2023). • Curiosity can improve job performance, creativity, adaptability, satisfaction, and well-being in organizational settings (Lievens et al., 2022; Horstmeyer, 2020; Wagstaff et al., 2021). • Domain-specificity may support curiosity in academic settings (Peterson & Cohen, 2019).
*Development*	• Understood as a metacognitive process, curiosity can be procedurally deployed (Goupil & Proust, 2023).

This review now turns to pinpointing specific aspects of curiosity that can be operationalized to support human flourishing. The conceptual framework is built around the following attributes from the synthesis matrix: (1) Dimensions: leveraging the multidimensional nature of curiosity; (2) Drive: emphasizing intentional curiosity as opposed to impulsive curiosity to encourage a deliberate decision to explore and learn; (3) Domains: prioritizing domain-general curiosity over domain-specific curiosity to apply across a wide range of cognitive, behavioral, and emotional contexts; (4) Development: focusing on curiosity as a cultivable skill rather than an innate trait to empower individuals to actively develop their curiosity. By focusing on these operational attributes of curiosity, there emerges an applied model that can be termed *systematic curiosity*—a multidimensional, intentional, domain-general, and cultivable form of curiosity (Table 2).

**Table 2:** Conceptual framework of systematic curiosity

Attributes	Characteristics
*Dimensions*	Multidimensional: Systematic curiosity can be directed towards exploring and understanding intellectual, emotional, or behavioral processes.
*Drive*	Intentional: Systematic curiosity is deliberate rather than impulsive, regardless of whether it is driven by intrinsic or extrinsic motivations.
*Domains*	Domain-General: Systematic curiosity is practically applicable in diverse real-world scenarios across educational, organizational, and therapeutic settings.
*Development*	Cultivable: While one’s innate curiosity may influence their baseline capacity to practice systematic curiosity, it is a skill that can be cultivated through training and development.

This conceptual framework offers a structured approach to leveraging curiosity for human flourishing. By emphasizing its multidimensional, intentional, domain-general, and cultivable attributes, systematic curiosity can support cognitive, emotional, and behavioral development. It can not only enhance our understanding of curiosity but also provide a foundation to devise practical strategies for fostering a more curious mindset across various domains, including educational, organizational, and therapeutic settings.

## Discussion

Based on a conceptual review covering seventy years of research, systematic curiosity is suggested as an applied model rooted in the multidimensional, domain-general, intentional, and cultivable aspects of curiosity. As a framework integrating cognitive, emotional, and behavioral components, systematic curiosity may serve as a mechanism to positively influence well-being and progress at the individual, group, and societal levels. This framework proposes to leverage curiosity as a practical tool for both personal and professional development, aligning it more effectively with the aim of supporting flourishing, and encouraging the personal exploration of various evidence-based activities so individuals can discover the most suitable strategies for their specific context and needs. In summary, systematic curiosity is suggested as a proactive tool for learning and engagement, rather than a reactive, instinct-driven response; a skill that can be developed and applied to promote flourishing.

While the conceptualization of systematic curiosity as proposed here is new, similar concepts can be found in various schools of thought throughout history. From ancient philosophy to modern psychology and neuroscience, constructs similar to systematic curiosity consistently emerge as foundational to holistic human development (Haynes, 2009; Odman & Govender, 2020). Most famously, the Socratic method involved a systematic approach of asking and answering questions to probe deeper understanding and encourage critical thinking (Gregory, 1983). Confucius emphasized the systematic pursuit of knowledge and moral self-cultivation (Kidd, 2018). Descartes also promoted a systematic method of doubting knowledge to establish a more certain foundation for science and philosophy—in fact, the full version of *cogito, ergo sum* is *ubito, ergo sum, vel, quod idem est, cogito, ergo sum*, which can be translated to “I doubt, therefore I am—or what is the same—I think, therefore I am” (Charles & Tannery, 1901). Lev Vygotsky’s (1978) theory of cognitive development includes the concept of the ‘Zone of Proximal Development’, which involves scaffolding learning experiences just beyond a child’s current ability, systematically fostering curiosity and learning.

**Fig. 1:**
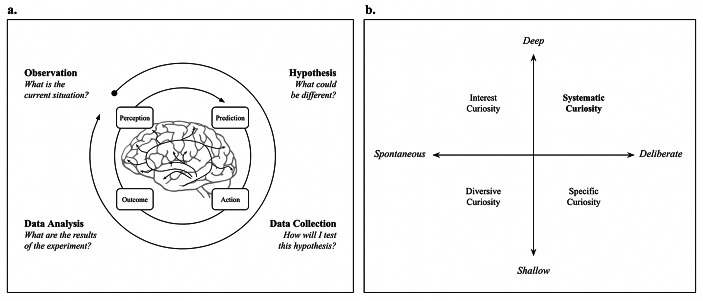
**(a)** A visual representation of deliberate self-inquiry through experimentation. **(b)** Typology of systematic curiosity in relation to related constructs. Copyright 2024 by Ness Labs. Reproduced with permission

Similar to scientific inquiry, systematic curiosity involves systematically confronting experiences, whether positive or negative, in a methodical manner driven by curiosity; questioning and exploring both internal experiences and external events; taking notes to notice patterns; forming hypotheses and testing assumptions (Jirout, 2020; Kuhn, 2004; Lindholm, 2018; Morris et al., 2012). Deliberate self-inquiry mirrors the experimental cycle of observing, hypothesizing, testing, and analyzing to refine predictions and adapt actions based on new evidence (Fig. 1a), akin to the action loops central to the cybernetics theory of self-regulation and the brain’s perception-action cycle (Carver & Scheier, 2012; Fuster, 2002; Kolb, 1971).

Situating systematic curiosity alongside other constructs of curiosity can help understand its potential value within the wider context of human flourishing. To that end, Fig. 1b illustrates how systematic curiosity can be positioned as “deep-deliberate” curiosity in relation to other forms of curiosity such as interest curiosity (“deep-spontaneous”, e.g., what is colloquially known as *falling into a wiki rabbit hole*), diversive curiosity (“shallow-spontaneous”, e.g., scrolling through social media), and specific curiosity (“shallow-deliberate”, e.g., quickly looking up information during a conversation to clarify a point).

**Fig. 2:**
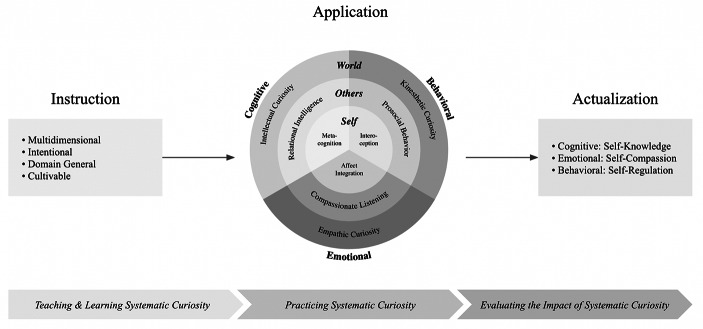
Operational model of systematic curiosity. Copyright 2024 by Ness Labs. Reproduced with permission

Furthermore, systematic curiosity can be understood as a mindset, the feelings that accompany it, and the set of actions that follow from this stance, thus encompassing three key components of human experience: cognitive, emotional, and behavioral. Within this framework, each of these components interact with three distinct levels: the self, the others, and the world. The first level (self) focuses on metacognition, affect integration, and interoception (Goupil & Proust, 2023; Kim et al., 2023; Leonard & Harvey, 2007; Price & Hooven, 2018; Solbakken et al., 2011); the second level (others) includes relational intelligence, compassionate listening, and prosocial behavior (Dunfield, 2014; Ekman, 2007; Miller, 2007); the third level (world) encompasses intellectual, emotional, and kinesthetic curiosity (LaBar et al., 2000; Russell, 2013; Vogl et al, 2020; Zhou et al., 2020). This layered approach provides a comprehensive model for visualizing how systematic curiosity could influence various facets of human experience, ranging from narrower curiosity at the individual level all the way up to broader curiosity at the societal level (Fig. 2).

Systematic curiosity might indirectly support factors associated with flourishing, such as social contribution and environmental mastery. For instance, sharing novel experiences and knowledge with others and building relationships through mutual self-disclosure has been found to facilitate social interactions (Kashdan & Roberts, 2006). Disseminating newfound information more broadly can also enhance one’s reputation and influence, providing external rewards for indulging curiosity (Kashdan et al., 2013; Kawamoto & Hiraki, 2017). This might explain why systematic curiosity—defined as an intellectual stance—is considered a character virtue in science (Pennock, 2019).

On the other hand, systematic curiosity might support flourishing by being an end in itself. Positive affect—a pleasant subjective feeling associated with positive emotions, moods, and sentiments—is an important factor of flourishing (Fredrickson & Losada, 2005), and the process of methodically exploring, questioning, and learning can be enriching on its own, contributing to psychological, emotional, and social well-being without needing to serve an external goal (Kashdan et al., 2009; Szumowska & Kruglanski, 2020). Systematic curiosity, therefore, may act as a bridge between the two perspectives regarding the teleological nature of curiosity (Szumowska & Kruglanski, 2020). It acknowledges the utility of curiosity as a means to achieve other ends, while recognizing the value of curiosity as intrinsically fulfilling.

Finally, systematic curiosity could also be applied to inquire about flourishing itself. A variety of activities have been found to promote flourishing, including cognitive activities such as practicing gratitude, savoring life’s experiences, and envisioning one’s ideal future self (VanderWeele, 2020), and behavioral activities such as performing acts of kindness (Kerr et al., 2015; VanderWeele, 2020). Furthermore, several workbook interventions targeting psychological distress have shown promise in contributing to overall flourishing (VanderWeele, 2020). The diversity of evidence-based activities suggests that there is no one-size-fits-all solution for flourishing, and individual differences may play a crucial role in determining the effectiveness of each activity (Jung, 2019; Keyes et al., 2015; Schotanus-Dijkstra et al., 2016; Wȩziak-Białowolska et al., 2019). Therefore, it might be beneficial to approach these potential strategies with systematic curiosity. By methodologically experimenting with various approaches, individuals can exert their sense of agency and discover the most effective methods for their unique needs and circumstances (Narasimhan et al., 2019; Shankar et al., 2019; Welzel & Inglehart, 2010). Satisfying their need for autonomy might even increase curiosity, creating a virtuous cycle (Schutte & Malouff, 2019).

In summary, systematic curiosity aims to offer a framework for supporting flourishing particularly in areas where personal agency plays a pivotal role. This framework promotes a more methodical approach to personal and professional development, encouraging individuals to examine their thoughts, emotions, and behaviors in a deliberate manner to better understand the world, others, and themselves.

The framework has several implications for theory and practice. In education, this model could inform the development of curricula and teaching methods that encourage students to explore topics methodically and inquisitively, nurturing their capacity to engage with new information in a deep and meaningful way (Scott-Barrett et al., 2023; Singh & Manjaly, 2022). At work, fostering a culture of systematic curiosity could involve training programs and policies that encourage employees to adopt a methodical approach to problem-solving and creativity, with the aim of improving productivity, innovation, and employee satisfaction (Chang & Shih, 2019; Kashdan et al., 2020; Lievens et al., 2022; Silva & Silva, 2023). In therapeutic settings, systematic curiosity aligns with and could potentially enrich approaches such as Cognitive Behavioral Therapy (CBT), mindfulness-based therapies, and other positive psychology interventions, offering a framework for helping clients explore and understand their thoughts, emotions, and behaviors more methodically, potentially contributing to improved mental health outcomes (Clark & Egan, 2015; Zainal & Newman, 2022).

While the proposed model holds promise for operationalizing curiosity as a tool to promote human flourishing, it is important to note its limitations. The conceptual framework was developed based on a comprehensive and methodological review (Jabareen, 2009) integrating multiple streams of research on curiosity and its connections to flourishing. However, the last steps in building a conceptual framework are validating and rethinking the framework (Jabareen, 2009). As such, systematic curiosity requires empirical testing and validation through quantitative and qualitative studies across diverse contexts (Creemers & Kyriakides, 2018; Lucas, 2003; Stenner & Smith III, 1982).

One potential direction for future research is clarifying the relationship between systematic curiosity and associated constructs such as wonder and intrinsic motivation (Ainley, 2019; Donnellan et al., 2022; Schinkel et al., 2023; Tang et al., 2022). Neuroscientific research could possibly help untangle some of these adjacent constructs (Hidi & Renninger, 2019). As uncertainty has been found on one hand to increase curiosity but to decrease happiness (van Lieshout et al., 2021) and on the other hand to be more easily managed with curiosity (Horstmeyer, 2020), research could also examine the interactions between uncertainty and systematic curiosity. Moreover, a compelling area of research would be to examine the interplay between neurodiversity, systematic curiosity, and distraction. Given the unique ways in which individuals with diverse neurocognitive profiles experience and express curiosity and distraction (Hilton et al., 2019; Sedgwick et al., 2019; Sonuga-Barke & Kostyrka‐Allchorne, 2023), exploring how systematically applying curiosity across all levels of the framework might help regulate distraction in neurodiverse populations could offer valuable insights for developing adaptive instructional strategies. Lastly, a prospective area of research would entail developing and testing instructional interventions that teach the cognitive, emotional, and behavioral dimensions of systematic curiosity across various cross-cultural settings (Schutte & Malouff, 2023).

While grounded in existing evidence on curiosity, the framework first and foremost serves as an initial foundation for future research. Empirical work is essential to evolve this conceptual framework into an evidence-based tool to support flourishing. Ultimately, the proposed framework invites researchers to consider curiosity’s potential role in flourishing and lays the groundwork for more targeted strategies to support holistic human development.

## Conclusion

This review reveals an evolving understanding of curiosity, moving beyond unidimensional perspectives to recognize curiosity’s multifaceted nature. It is argued that operationalizing curiosity to promote flourishing requires focusing on its multidimensional, intentional, domain-general, and cultivable features. As a framework integrating cognitive, emotional, and behavioral components, systematic curiosity is suggested as a holistic model to positively influence well-being and growth at the individual, group, and societal levels. The possible applications of systematic curiosity extend beyond personal development, with the potential to foster a culture of deliberate inquiry that can in turn lead to innovative problem-solving, enhanced social cohesion, and a deeper understanding of diverse perspectives. By encouraging methodical questioning, exploration, and learning, systematic curiosity offers a pathway toward flourishing. Its broad applicability and potential impact requires further empirical research to explore its effectiveness and refine its application.

## Electronic supplementary material

Below is the link to the electronic supplementary material.

Supplementary Material 1
