# Supplementary material for: Systematic Curiosity as an Integrative Tool for Human Flourishing: A Conceptual Review and Framework
Source: Integr Psychol Behav Sci. 2024 Jul 9;58(4):1876–94. doi: 10.1007/s12124-024-09856-6 (PMC11638310; doi:10.1007/s12124-024-09856-6)
Supplement: Supplementary file 1 — Supplementary Material 1 [file 12124_2024_9856_MOESM1_ESM.pdf]

Appendix 1. Search Strategy for Web of Science

(TI=(curiosity) AND (TS=(framework OR model OR theor* OR conceptual* OR operational OR "operational definition"OR construct OR paradigm OR schema OR measurement OR scale OR assessment OR instrument)

Appendix 2. Search Results after Screening

ID	Title	First Author	Year	Journal	DOI
1	A Theory of Human Curiosity	Berlyne, D.	1954	British Journal of Psychology	10.1111/j.2044-8295.1954.tb01243.x
2	Effect of Fear And Anxiety On Exploration And Curiosity - Toward A Theory Of Exploration	Lester, D.	1968	Journal of General Psychology	10.1080/00221309.1968.9710458
3	Critical-Review of State-Trait Curiosity Test Development	Boyle, GJ.	1983	Motivation and Emotion	10.1007/BF00991647
4	Curiosity and Exploration - Theories and Results	Voss, HG.	1983	Academic Press	10.1016/C2013-0-11659-5
5	The Psychology of Curiosity - A Review and Reinterpretation	Loewenstein, G.	1994	Psychological Bulletin	10.1037/0033-2909.116.1.75
6	Curiosity and the Pleasures of Learning: Wanting and Liking New Information	Litman, JA.	2005	Cognition & Emotion	10.1080/02699930541000101
7	Curiosity and Medical Education	Dyche, L.	2011	Medical Education	10.1111/j.1365-2923.2011.03944.x
8	Children's Scientific Curiosity: In Search of an Operational Definition of an Elusive Concept	Jirout, J.	2012	Developmental Review	10.1016/j.dr.2012.04.002
9	Intellectual Curiosity a Principle-Based Concept Analysis	Russell, BH.	2013	Advances in Nursing Science	10.1097/ANS.0b013e3182901f74
10	Information-Seeking, Curiosity, and Attention: Computational and Neural Mechanisms	Gottlieb, J.	2013	Trends in Cognitive Sciences	10.1016/j.tics.2013.09.001
11	The Psychology and Neuroscience of Curiosity	Kidd, C.	2015	Neuron	10.1016/j.neuron.2015.09.010
12	Intrinsic Motivation, Curiosity, and Learning: Theory and Applications in Educational Technologies	Oudeyer, PY.	2016	Motivation: Theory, Neurobiology And Applications	10.1016/bs.pbr.2016.05.005
13	Disentangling Curiosity: Dimensionality, Definitions, and Distinctions from Interest in Educational Contexts	Grossnickle, EM.	2016	Educational Psychology Review	10.1007/s10648-014-9294-y
14	On Curiosity: A Fundamental Aspect of Personality, a Practice of Network Growth	Zurn, P.	2018	Personality Neuroscience	10.1017/pen.2018.3
15	How Curiosity Enhances Hippocampus-Dependent Memory: The Prediction, Appraisal, Curiosity, and Exploration (PACE) Framework	Gruber, MJ.	2019	Current Opinion in Behavioral Sciences	10.1016/j.tics.2019.10.003
16	Process Account of Curiosity and Interest: A Reward-Learning Perspective	Murayama, K.	2019	Educational Psychology Review	10.1007/s10648-019-09499-9
17	A Case for Domain-Specific Curiosity in Mathematics	Peterson, EG.	2019	Educational Psychology Review	10.1007/s10648-019-09501-4
18	The Generative Role of Curiosity in Soft Skills Development for Contemporary Vuca Environments	Horstmeyer, A.	2020	Journal of Organizational Change Management	10.1108/JOCM-08-2019-0250
19	Measures of Curiosity: A Literature Review	Wagstaff, MF.	2021	Human Resource Development Quarterly	10.1002/hrdq.21417
20	Killing the Cat? A Review of Curiosity at Work	Lievens, F.	2022	Academy of Management Annals	10.5465/annals.2020.0203
21	Curiosity-Driven Exploration: Foundations in Neuroscience and Computational Modeling	Modirshanechi, A.	2023	Trends In Neurosciences	10.1016/j.tins.2023.10.002
22	Curiosity as a Metacognitive Feeling	Goupil, L.	2023	Cognition	10.1016/j.cognition.2022.105325
